# Supplementary material for: Plasma Bead Entrapped Liposomes as a Potential Drug Delivery System to Combat Fungal Infections
Source: Molecules. 2022 Feb 7;27(3):1105. doi: 10.3390/molecules27031105 (PMC8840493; doi:10.3390/molecules27031105)
Supplement: Supplementary file 1 [file molecules-27-01105-s001.zip › molecules-1540030-supplementary.pdf]

molecules

MDPI

Supplementary Materials

Plasma Bead Entrapped Liposomes as a Potential Drug Delivery System to Combat Fungal Infections

Munazza Tamkeen Fatima [1,*,†], Zeyaul Islam [2,‡], Ejaj Ahmad [1,§], Mehboob Hoque [1] and Marriam Yamin [3,‖]

1 Interdisciplinary Biotechnology Unit (IBU), Aligarh Muslim University (AMU) Aligarh, Uttar Pradesh—202002, India.
2 Laboratório Nacional de Biociências (LNBio), Centro Nacional de Pesquisa em Energia e Materiais (CNPEM), Campinas, Sao Paulo, 13083-970, Brazil.
3 Functional and Molecular Biology, Biochemistry, UNICAMP, Campinas, Sao Paulo, 13083-970, Brazil;
† Current address: Department of Neurosciences, Hamad Medical Corporation, Qatar.
‡ Current address: Qatar Biomedical Research Institute, Hamad Bin Khalifa University, Qatar Foundation, P.O.Box 5825 Doha, Qatar.
§ Current address: Central Molecular Laboratory, Govind Ballabh Pant Institute of Postgraduate Medical Education and Research (GIPMER), New Delhi, India. 110002
‖ Current address: Applied Bio-Chemistry Lab, Department of Biosciences, Aliah University, Kolkata 700156, India
* Correspondence: munazza.ibu@gmail.com

Table S1. Physicochemical properties of the entrapped pharmaceuticals.

Molecules	Molecular Weight	Solubility	Chemical Structure	Use
Amphotericin B	924.1 g/mol	Soluble in DMSO upto 30-40 mg/ml	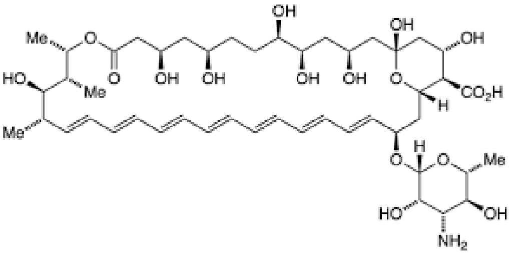	Drug-Antifungal agent
Blue dextran	~2000 kDa	Soluble in water upto 50 mg/ml	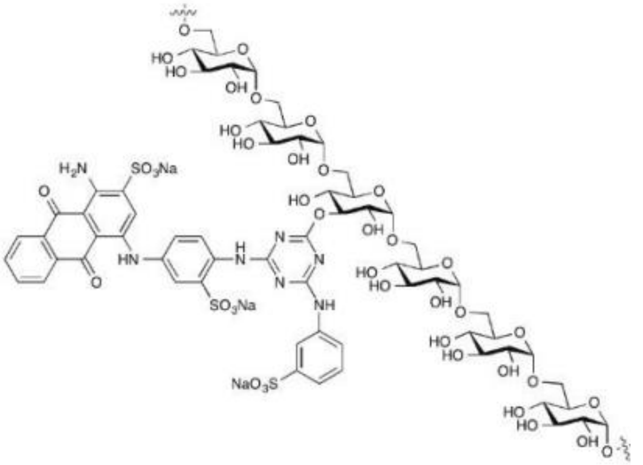	Molecular marker
HRP	~44kDa	Readily soluble in water	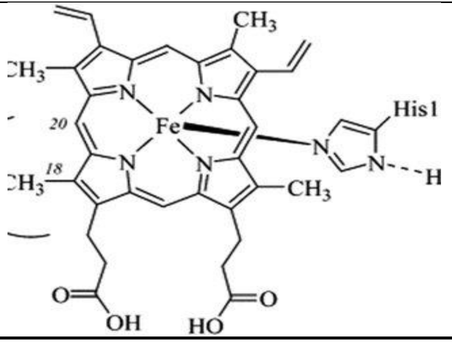	Enzyme-Biochemical applications
